# Hypoglycemia as a Manifestation of Shapiro Syndrome

**DOI:** 10.7759/cureus.23120

**Published:** 2022-03-13

**Authors:** Connor Lewis, Natasha Freeman, Neil Gupta

**Affiliations:** 1 Internal Medicine, Yale School of Medicine, New Haven, USA; 2 Internal Medicine, Yale New Haven Hospital, New Haven, USA

**Keywords:** blood glucose, diaphoresis, hypoglycemia, hypothermia, shapiro syndrome

## Abstract

Shapiro syndrome is an extremely rare disorder of dysautonomia characterized by paroxysmal episodic hypothermia to below 95°F. Many patients with Shapiro syndrome improve with medical management, though a minority of cases are refractory to treatment. Our patient with adult-onset Shapiro syndrome is an atypical case. Our patient has been refractory to medical treatment as well as chemical sympathectomy. Based on a review of the literature, this is also the first reported case of hypoglycemia with Shapiro syndrome episodes in the absence of other metabolic diseases. This case suggests that hypoglycemia could be a potential manifestation of Shapiro syndrome.

## Introduction

Shapiro syndrome is an extremely rare disorder of dysautonomia characterized by paroxysmal episodic hypothermia to below 95°F. In 2014, a review paper on the topic recorded 52 cases across 34 publications after extensive literature review from the years 1934-2013 [1]. A range of signs and symptoms have been described related to Shapiro syndrome, including agenesis of the corpus callosum, pallor, hyperhidrosis, and altered consciousness [1]. The pathophysiology underlying Shapiro syndrome is unknown, though mechanisms have been proposed regarding the corpus callosum and hypothalamus [1,2]. Many patients with Shapiro syndrome improve with medical management, though a minority of cases are refractory to treatment. We present a unique case of adult-onset Shapiro syndrome refractory to medical treatment as well as sympathectomy. To our knowledge, this is one of the first reported cases of recurrent hypoglycemia associated with dysautonomic episodes.

## Case presentation

Our patient is a 51-year-old male who presented to the emergency room one day after being discharged from a prior autonomic crisis. He was noted to have a long-standing history of Shapiro syndrome without agenesis of the corpus callosum since at least 2014. He had multiple hospitalizations, with trials of cyproheptadine, clonidine, carbamazepine, glycopyrrolate, and acetazolamide. All of these medications have been ineffective in our patient despite their success in other Shapiro syndrome cases. He had also been treated with levetiracetam and pregabalin, without difference in the timing or severity of his episodes.

During his most recent presentation, he was dyspneic, hypothermic to 93.4°F, and hypotensive to 76/57 mmHg, and had difficulty in speaking due to frequent moaning. He required admission to the intensive care unit for management of his autonomic crises. He was treated with a levetiracetam 2.5 gram loading dose following the onset of a crisis, followed by levetiracetam 500 milligrams twice daily, as well as acetazolamide 500 milligrams twice daily and pregabalin 25 milligrams three times daily for pain. These medications had no effect on the frequency or duration of his episodes. He was also given norepinephrine, midodrine, and fluid boluses for his hypotension, without hemodynamic improvement.

**Figure 1:**
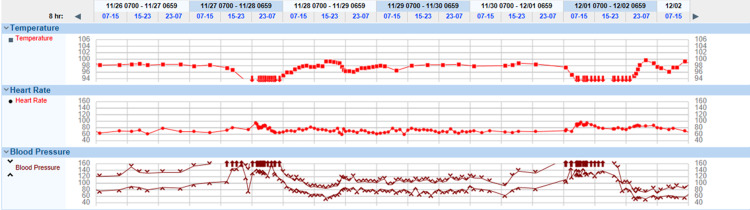
Patient’s vitals during two Shapiro syndrome crises These graphs demonstrate the patient’s temperature, heart rate, and blood pressure over a six-day period where the patient suffered two dysautonomic episodes involving initial hypertension and hypothermia followed by hypotension and normothermia.

**Figure 2:**
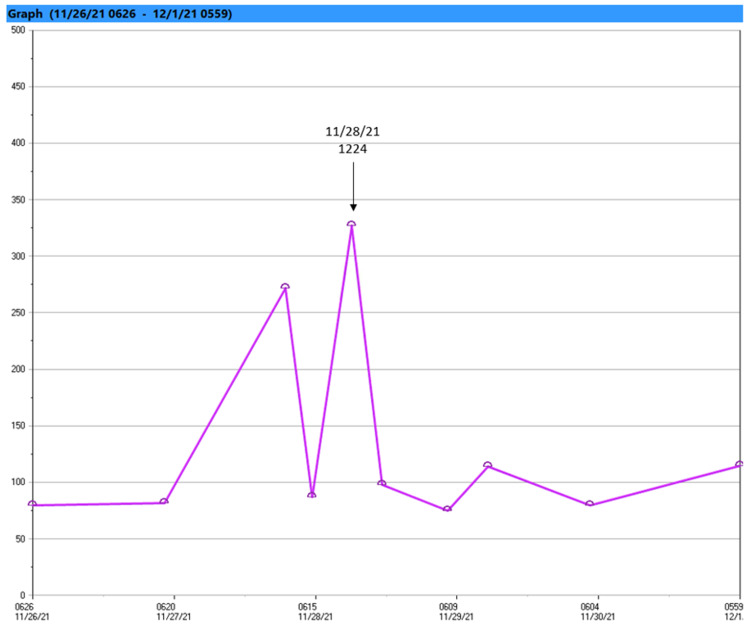
Patient’s blood glucose during a Shapiro syndrome crisis This graph demonstrates the patient’s blood glucose during the first crisis in Figure 1; the labeled spike in blood glucose indicates the patient being treated for hypoglycemia with dextrose 50% in water.

Over the next three days, his hemodynamics and temperature finally improved, and our patient was transferred from the intensive care unit to the floor. Soon thereafter, he had another crisis, where he became hypothermic to 81.2°F and hypertensive to 235/128 mmHg. After returning to normal temperature the next morning, he became hypotensive to 78/44 mmHg. Notably, this hypotension did not correct despite fluid administration. The patient refused vasopressors and slowly recovered. Several days later, he had yet another episode where he became extremely diaphoretic, hypertensive to 237/145 mmHg, and hypothermic to 96.7°F. He was also acutely agitated, attempting to get out of bed despite restraints. At that point, he was transferred to a higher level of care in the Step-Down Unit (SDU). Since admission to the SDU, he suffered 14 major autonomic crises in a 63-day period. The longest period between crises was 10 days, with the shortest period between crises being two days. Figures 1, 2 outline our patient's vitals and blood glucose, respectively, over a 6-day period that is representative of the timeline of most of his crises.

**Figure 3:**
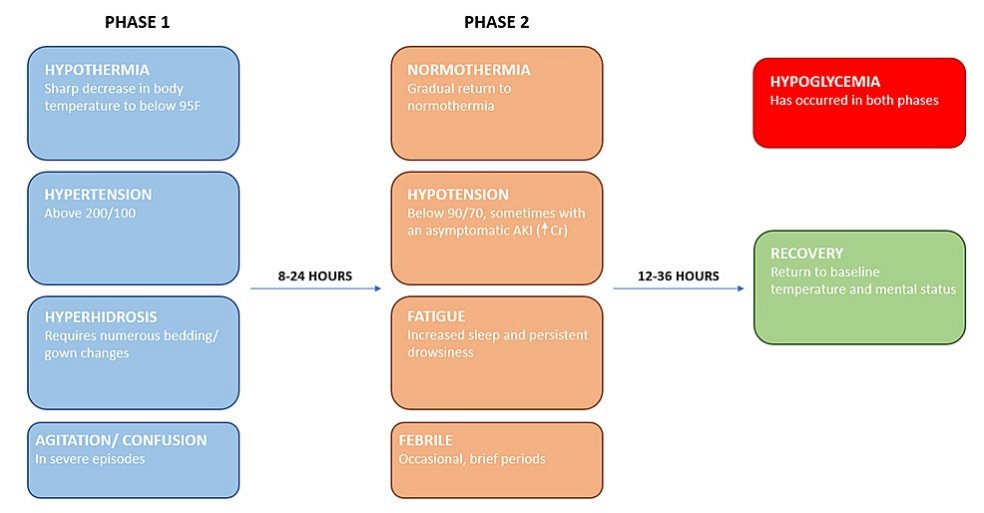
Patient’s symptoms during a Shapiro syndrome crisis This flow chart outlines a Shapiro syndrome crisis as experienced by our patient.

His crises were noted to have two phases, defined by a set of symptoms outlined in Figure 3. Symptomatic hypoglycemia was noted as a consistent component of his crises, with a blood glucose level between 40 and 60. This hypoglycemia was effectively treated with intravenous dextrose.

After one crisis, where the patient’s blood glucose was noted to be in the 40s, hypoglycemia labs were drawn. The patient was noted to have a serum glucose of 45 mg/dL, insulin level of 5.5 μU/mL (normal: 2.6-24.9 μU/mL), C-peptide level of 4.3 ng/mL (normal: 1.1-4.4 ng/mL), proinsulin level of 6.3 pmol/L (normal: <18.8 pmol/L), and a beta-hydroxybutyrate level of 0.21 mmol/L (normal: ≤0.27 mmol/L). The insulin, proinsulin, and C-peptide levels were unexpectedly within a normal range, though they should have been decreased given his low blood glucose. AM and PM cortisol levels were taken to rule out adrenal insufficiency, revealing an AM cortisol level of 7.5 μg/dL and PM cortisol level of 4.7 μg/dL, both within normal limits. He was found to have an insulin-like growth factor 1 level of 177 ng/dL (normal: 52-328 ng/dL). Our patient’s 24-hour urine metanephrine and normetanephrine have been consistently elevated, with urine metanephrine of 215-441 μg (normal: 24-96 μg) and normetanephrine of 565-1,112 μg (normal: 82-500 μg) with three different measurements over a span of two months. Carnitine levels of our patient were assessed, with a free carnitine of 28.8 uM (normal: 24.0-66.0 uM) and an esterified carnitine of 7.9 uM (normal: 4.0-32.0 uM), with a carnitine, esterified/free ratio of 0.3 (normal: 0.1-0.9 uM). CT of the abdomen did not reveal any adrenal or pancreatic masses. Brain MRI revealed no hypothalamic masses or pituitary abnormalities.

Symptomatic management of his crises focused on alleviating his hypertension, hypotension, hypothermia, agitation, and hypoglycemia. All symptomatic management failed except for hypoglycemia treatment with dextrose and treatment of agitation with haloperidol. Both his hypertension and hypotension have been extremely resistant to fluids and other medications. Long-acting anti-hypertensives, such as labetalol and hydralazine, have not been pursued due to long-term effects and worsening rebound hypotension. His hypothermia has been unaffected by treatment with Bair Hugger (3M, St. Paul, MN) and warm IV or PO fluids. Despite treatment measures, he has continued to have recurrent crises, affecting his ability to be safely discharged home or to a long-term care facility.

Given his lack of response to medical treatments, a continuous sympathetic blockade was attempted prior to his current hospitalization to determine if total sympathectomy could be curative. Sympathectomy has been proposed as a potential treatment for periodic hypothermia and hyperhidrosis, with at least one recorded case of successful treatment in a Shapiro syndrome patient [3,4]. He underwent a bilateral video-assisted thoracic surgery (VATS) for the placement of subpleural pain catheters along his sympathetic chain. Bupivacaine 0.5% infusion was started at 4 mL/hour to induce continuous sympathetic blockade. Unfortunately, he had recurrent autonomic crises despite this treatment. Total sympathectomy was not pursued following this outcome.

## Discussion

Based on a review of the literature, this is the first reported case of hypoglycemia with Shapiro syndrome episodes in the absence of other metabolic diseases. We found one reported case of hypoglycemia with Shapiro syndrome in a 39-year-old female, but this patient was also diagnosed with carnitine deficiency, which is a known cause of hypoglycemia [5]. Notably, our patient had carnitine levels within normal limits.

Etiologies of hypoglycemia were ruled out through diagnostic testing. The lack of significantly elevated insulin and proinsulin in the context of normal CT without pancreatic masses ruled out an insulinoma. The normal levels of AM and PM cortisol ruled out adrenal insufficiency. The normal insulin-like growth factor 1 level ruled out other endocrine etiologies that could cause hypoglycemia. Based on imaging, there was a lack of evidence to support the presence of tumors that could be causing secretion of insulin or antibodies to insulin that would explain hypoglycemia. There was lack of evidence of medications, such as sulfonylureas, that would explain hypoglycemia, and the patient was noted to have adequate nutritional intake without evidence of ketosis to explain potential hypoglycemia related to dietary intake. He had no history of diabetes. Our case is an example of unexplained hypoglycemia connected to our patient’s Shapiro syndrome pathophysiology, which is noted to occur only in the context of his Shapiro crises.

These hypoglycemic symptoms are likely exacerbating our patient’s Shapiro syndrome due to the effects of hypoglycemia on the central nervous system. Episodes of profound hypoglycemia are known to cause structural and functional disturbances in the brain. Glucose levels of less than 3 mmol/L (54.6 mg/dL) can cause seizures and loss of consciousness, and recurrent hypoglycemic episodes can damage brain matter and lead to a decline in cognitive function [6]. These findings are especially concerning for our patient because his syndrome may be due to dysfunction in the hypothalamus and other deep brain structures. Damage to these and other areas of our patient’s brain could decrease his mental reserve and may further exacerbate his symptoms in unforeseen ways. If our patient were to suffer an episode with profound hypoglycemia outside a hospital setting, there is a risk that he may develop seizures that would be difficult to distinguish from the normal muscle stiffness and violent shivering that occur with his hypothermic episodes.

In another case, a 58-year-old male presented with the classic symptoms of Shapiro syndrome but with a pathophysiology solely due to hypoglycemia. This patient presented with diaphoresis and hypothermia resistant to local warming. Only after correction of his blood glucose did his hypothermia and diaphoresis improve. This case report indicated that hypothermia has previously been documented as an adaptive neuroprotective mechanism in people with acute hypoglycemia [7]. This case shares many similarities with our patient’s presentation of Shapiro syndrome: resistant hypothermia and diaphoresis. However, our patient’s presentation differs in that our patient does not always start his crises with a low blood glucose, and symptoms can persist for many hours even after blood glucose correction. However, the shocking similarity in initial patient presentation between these two cases, and the known neurotoxic effects of hypoglycemia on the central nervous system, imply that there is a mechanistic connection between the hypothermic, diaphoretic crises of Shapiro syndrome and hypoglycemia that warrants further research.

Our patient’s syndrome has been refractory to all medications and even chemical sympathectomy. This is an uncommon finding since a previous review of Shapiro Syndrome cases found that only nine of the 52 reviewed cases were unresponsive to treatment [1]. Among our patient’s symptoms, hypoglycemia is the most unique and one of the more concerning symptoms. His hypothermic episodes do not seem to be life-threatening and have been successfully managed with blankets alone. However, a significant drop in blood glucose could have detrimental effects on our patient’s health and central nervous system and must be adequately managed before he can be discharged. Dextrose infusions and warm PO fluids with sugar have been successful in acutely treating this hypoglycemia. However, our patient’s blood glucose levels will have to be continually monitored during his episodes and represent an additional complication related to his Shapiro syndrome. The lack of effective treatment for his Shapiro crises has significantly impaired our patient’s quality of life, and, due to the unclear pathophysiology of this disease, he has remained hospital-bound.

## Conclusions

Our patient presents with an unusual case of Shapiro syndrome that has been refractory to all attempted medical treatment. His hypoglycemia is an especially unusual and novel presentation of Shapiro syndrome crises. Other metabolic diseases and medications that would cause hypoglycemia have been ruled out. Previous case reports have discussed patients who present with hypothermic, diaphoretic symptoms linked to hypoglycemia, but this mechanism has not been investigated in the context of Shapiro syndrome. The link between hypoglycemia and Shapiro syndrome is a potential area of future research about this rare disease.
